# A Novel Method for Developing Thin Resin Scintillator Screens and Application in an X-ray CMOS Imaging Sensor

**DOI:** 10.3390/s23146588

**Published:** 2023-07-21

**Authors:** Dionysios Linardatos, George Fountos, Ioannis Valais, Christos Michail

**Affiliations:** Radiation Physics, Materials Technology and Biomedical Imaging Laboratory, Department of Biomedical Engineering, University of West Attica, Ag. Spyridonos, 12210 Athens, Greece; dlinardatos@uniwa.gr (D.L.); gfoun@uniwa.gr (G.F.); valais@uniwa.gr (I.V.)

**Keywords:** CMOS sensors, X-ray, Gd_2_O_2_S:Tb, non-destructive testing, DQE, scintillators, epoxy resin, sedimentation, graphite, 3D printing

## Abstract

Scintillating screens for X-ray imaging applications are prepared with various methods. Among them, the classic sedimentation method presents certain weak points. In this context, a novel fabrication process was developed that offers simplicity, economy of resources and time, while the screens exhibit adequate durability and image quality performance. The proposed technique involves a resin mixture that contains the phosphor in powder form (Gd_2_O_2_S:Tb in the present work) and graphite. The novel method was optimized and validated by coupling the screens to a complementary metal oxide semiconductor (CMOS) X-ray sensor. Indicatively, screens of two surface densities were examined; 34 mg/cm^2^ and 70 mg/cm^2^. Various established image quality metrics were calculated following the IEC 62220-1 international standard, including the detective quantum efficiency (DQE). Comparisons were carried out under the same conditions, with a sedimentation screen reported previously and a screen of wide commercial circulation (Carestream Min-R 2190). The novel screens exhibit has comparable or even better performance in image-quality metrics. The 34 mg/cm^2^ screen achieves a DQE 15–20% greater than its comparison counterpart, and its limiting resolution was 5.3 cycles/mm. The detector coupled to the 70 mg/cm^2^ screen achieved a DQE 10–24% greater than its own counterpart, and its limiting resolution was found to be 5.4 cycles/mm.

## 1. Introduction

In X-ray medical radiography, a great part of the radiation detector is based on the combination of a scintillator with an optical sensor, both in the sector of diagnostic radiology (classic radiography, mammography, fluoroscopy, computed tomography), as well as in the sector of nuclear medicine (gamma camera, positron-emission tomography). In this sort of equipment, scintillators are found in the form of monocrystals, ceramic coatings, in a columnar form, or as a thin layer of phosphor material in granular form. Scintillators are coated on, or coupled to optical sensors that can either be complementary metal oxide semiconductors (CMOS), charge-coupled devices (CCD), a-Si/TFT, photodiode arrays, photocathodes, or simply films [1,2].

In medical imaging, it is always desired to achieve the extraction of the optimum image quality using the lowest attainable radiation dose burden for the patient. Accordingly, the interest of the scientific community is always increased for the development of novel detectors that combine the desired characteristics [3,4]. Furthermore, the advent of new diagnostic techniques demands that the available high performance scintillators fulfil specific criteria [5,6,7,8].

A crucial parameter for the total efficiency of a detector is the amount of energy absorbed within its scintillator mass. The density and its effective atomic number greatly influence the radiation absorption efficiency. This in turn affects the signal to noise ratio (SNR) { XE “signal to noise ratio:SNR” } and consequently the radiation dose that has to be delivered to the patient [9,10,11,12]. Other desired characteristics include the following: ruggedness and absence of hygroscopicity; low levels of afterglow; adequate homogeneity in the distribution of the emission centers (activator sites–impurities) within the phosphor mass, low manufacturing cost and complexity in the production process [6,13,14]. In achieving the desired characteristics, much importance is given to the development of deposition methods in a screen format beyond the established techniques and the investigation of how the various parameters affect the resulting image quality.

Currently, scintillator screen manufacturing methods can be overly complicated and costly to run. Processes involve strict preparation conditions and long production cycles, while the results are sometimes delicate and prone to mechanical or environmental damage [15,16,17,18,19]. A simple technique targeted to limited productions, research settings and sites lacking specialized instrumentation is the classic sedimentation method [20]. It requires equipment typical for a scientific laboratory. Yet, the resulting screens exhibit reduced mechanical strength. The fused silica or borosilicate glass substrate that is mainly used for the scintillator visible light propagation is not flexible and the phosphor remains exposed to external factors. Moreover, the binding substance loses its cohesive properties after a period of time and the phosphor returns to its initial form: loose powder. By concept, the sedimentation method involves the use of large amounts of phosphor material in order to achieve adequate homogeneity. The excess material is wasted [20].

In this context, a novel screen fabrication process was developed that does not require complicated instrumentation, while at the same time it addresses the problem of the amount of wasted phosphor. It also enhances the product’s lifespan. The aforementioned objectives were pursued while aiming at not sacrificing the imaging properties of the produced screens, but rather achieving comparable performance to screen samples that were prepared by the classic sedimentation method or even screens of wide commercial use.

Inspired from the classic sedimentation, the proposed technique involves a resin mixture containing the phosphor powder and a small amount of graphite. Centrifugation forces the phosphor particles to condense at the lowest part of the screen and graphite to rest above this layer. The resulting screen is a tile of hardened resin in which the bottom “active” layer is a thin phosphor film.

The novel preparation method was developed and optimized by integrating the new screens in an X-ray detecting system based on a CMOS sensor. The image quality performance of the detector is assessed on the basis of widely-accepted criteria, following procedures standardized by the International Electrotechnical Commission (IEC). With the intention of ensuring valid comparisons with recent and earlier data alike, the IEC 62220-1:2003 standard (IEC 2003 for brevity) [21] is adopted throughout the validation and comparison of the present detector to previously published data.

## 2. Materials and Methods

The new screen preparation method is based on the concept that a host material encapsulates the active phosphor and provides structure and protection. This host material has to be transparent to the light emitted by the phosphor upon X-ray irradiation. The phosphor has to assume a thin-layer form in order for the produced image resolution to be acceptable. The instrumentation needed is rather common for a scientific laboratory and includes analytical scales, a centrifuge, a vacuum chamber and an ultrasound homogenizer.

The novel method is described in detail through the first parts of this section, along with the intermediate steps towards its consolidation. The subsequent parts of the section describe the methods used to validate the novel screen preparation procedure. These included the estimation of established image quality metrics such as the signal transfer property (STP), modulation transfer function (MTF), normalized noise power spectrum (NNPS) and detective quantum efficiency (DQE), according to international standards.

### 2.1. Overview of the Method

This process involves the use of a silicone mold that gives the desired shape to the working mixture. The mixture comprises the phosphor and graphite, both in granular form. These are incorporated within the resin–hardener combination that will act as an encapsulation and light propagation medium. The phosphor will give the screen its luminescence characteristics, while graphite will contribute towards reducing the blur caused by backscattered optical photons.

The working mixture is produced in sufficient quantity so that the resulting screen reaches a certain thickness for adequate mechanical strength. Phosphor and graphite powders are added. Upon centrifugation and due to the difference in their densities, the phosphor particles are condensed at the lowest part of the screen, while graphite rests above the phosphor layer. Centrifugation parameters and relative quantities have been optimized in order to achieve the desired result. With the components described so far, the resulting screen is a tile of ~2 mm thickness, where the bottom “active” layer is a thin phosphor film. This phosphor layer can be adapted to the desired thickness, from tens to hundreds of micrometers. The volume of the resin that rests above the supernatant has been proven as the minimum required for the screen’s mechanical stability. Other thicknesses are also possible, depending on the application.

**Figure 1:**
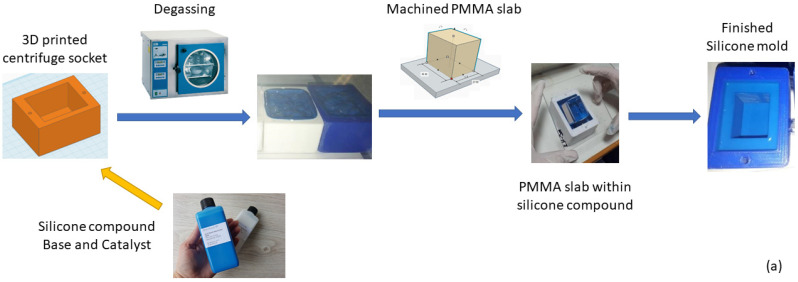

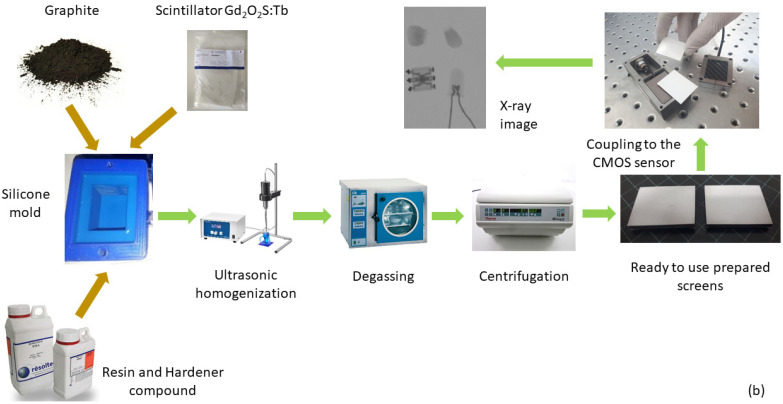
Schematic summary of the (**a**) silicone mold preparation (for multiple uses); (**b**) novel 5-step scintillator screen fabrication procedure. The outer centrifuge socket dimensions are approximately 80 × 60 × 40 mm^3^. The inner silicone mold dimensions are equal to the prepared screens and the resulting X-ray image, i.e., 36 × 27 mm^2^.

After centrifugation, the silicone mold with the phosphor–resin mixture remains at rest up to the complete hardening of the resin. The following are the fabrication steps of the silicone mold (which is for multiple uses), as well as the five steps of the novel screen preparation procedure. These are schematically presented in Figure 1a,b, respectively.

### 2.2. Silicone Mold Preparation

#### 2.2.1. PMMA Slab

Regarding the shaping of the silicone mold, a polymethyl methacrylate (PMMA) slab tailored for the specific application’s dimensions was used. In line with the CMOS sensor that will be used, the PMMA slab’s dimensions are 36 × 27 mm^2^.

#### 2.2.2. Centrifuge Socket

For accommodating the silicone mold, a special socket is constructed that fits in the centrifuge buckets described through the following steps of the procedure. The centrifuge used for the experiments was the benchtop Heraeus Multifuge 1S (Heraeus GmbH, Hanau, Germany).

#### 2.2.3. Silicone Compound Preparation

The silicone mold is prepared by measuring equal amounts (by weight) of Zhermack ZA 22 Mold Base and Catalyst (Zhermack SpA, Badia Polesine, Italy) [22] and mixing thoroughly for a minimum of 5 min. During mixing, air becomes trapped in the silicone. The next step of degassing in a vacuum chamber helps eliminate the air pockets. The silicone-filled sockets are placed in the chamber of a Selecta Vaciotem-T vacuum drying oven (JP Selecta, Abrera, Spain), where only the vacuum pump is activated, without heating being applied. The rotary-vane two-stage pump has a vacuum limit of 5 Pa. The silicone undergoes degassing for 5 min.

#### 2.2.4. Silicone Mold Cast

The PMMA slab is immersed in the silicone compound and kept steadily in place. The arrangement is kept steady up to the silicone’s complete hardening, i.e., for a minimum of 24 h. After the silicone is consolidated, the PMMA slab is retracted and the silicone mold is ready to be filled with the mixture of resin and scintillator powder. After the screen’s preparation, it can be reused multiple times.

### 2.3. Novel 5-Step Screen Fabrication Procedure

#### 2.3.1. Resin–Phosphor–Graphite Compound

The epoxy resin that will be part of the final product is prepared by thoroughly mixing the following components: WWA Resin and WWB4 Hardener (Resoltech SAS, Rousset, France) at a weight ratio 70:30. The components’ quantities are calculated so as to suffice for a final screen thickness of ~2 mm. The phosphor amount that has to be poured in the mixture depends on the desired surface density of the final screen. Indicatively, for a screen of 34 mg/cm^2^ surface density, one has to multiply with the screen’s surface, and, therefore, use 330 mg of phosphor powder. Surface density or coating weight is the mass of the phosphor particles per unit surface.

The scintillator Gd_2_O_2_S:Tb was used for the proposed method. It was obtained in the form of powder from Phosphor technology Ltd. (PTL grade UKL65/N-R1; Phosphor technology Ltd., Stevenage, UK) [23].

Another essential element of the novel method is the light-absorbing effect of a layer above the light-producing one. This is made of graphite particles. Synthetic graphite (SKU 282863; Merck KGaA, Darmstadt, Germany) was employed in the form of high-purity powder. The fact that the mixture components have suitable densities and properly selected amounts forces them to form layers at certain heights within the screen cross-section.

It is emphasized that literally the whole amount of the phosphor material is utilized with the proposed protocol. A significant improvement is achieved over the classic sedimentation technique where the material loss amounts to nearly 78% [24].

#### 2.3.2. Ultrasonic Homogenization

The final compound is homogenized for 2 min with the ultrasonic homogenizer Sonopuls HD 2200.2 (Bandelin Electronic GmbH, Berlin, Germany) fitted with the HF generator GM 2200, ultrasonic converter UW 2200, booster horn SH 213 G, and the titanium flat tip TT 13 that has a diameter of 13 mm. In order to avoid excessive heat build-up that could compromise the phosphor properties, the instrument is set at 10% of its maximum power which is 200 W.

#### 2.3.3. Degassing

The silicone mold is transferred to the vacuum chamber of the Selecta Vaciotem-T vacuum drying oven where it is degassed at room temperature for 5 min.

#### 2.3.4. Centrifugation

The settings that performed the best were 300 rpm for 2 min. The socket with the silicone mold and the resin–phosphor–graphite mixture was placed in a centrifuge bucket. Appropriate weight was added at the opposite side of the rotor as a counterweight.

#### 2.3.5. Consolidation

After centrifugation, the whole bucket with its contents was kept steady for as long as the epoxy hardening required, usually for a minimum of 72 h.

### 2.4. Prepared Screens

The screens’ target surface densities were selected to be 34 mg/cm^2^ and 70 mg/cm^2^ for this stage of the experiments. This combination is representative of general radiography and mammography applications [25,26,27]. It also permits the direct comparison to previously fabricated screens with the sedimentation method, as well as commercial ones such as the Carestream Min-R 2190 [28].

**Figure 2:**
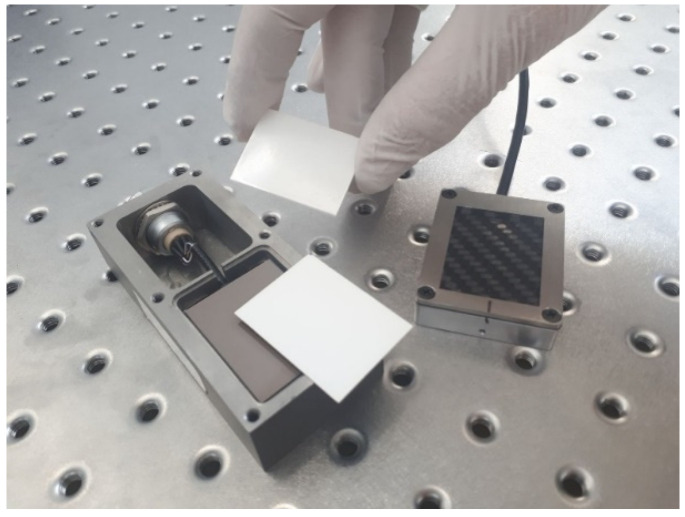
Preparation of the detector. The dimensions of the CMOS sensor’s active area and the prepared screens are 36 × 27 mm^2^.

The resulting scintillating screen under investigation is directly coupled to the entrance of the above-mentioned CMOS photodiode array, with the phosphor layer in direct contact with the sensor’s surface. Thus, the resin mixture quantities were selected such that the resulting screens fit in the limited space of the CMOS module, i.e., 36 mm length, 27 mm width, ~2 mm thickness. A snapshot of this stage is illustrated in Figure 2.

### 2.5. Imaging Performance Assessment

#### 2.5.1. Irradiation Conditions and Geometry

The medical X-ray radiography setting is now considered. The image quality performance of the detector is assessed on the basis of widely-accepted criteria, following procedures standardized by the International Electrotechnical Commission (IEC).

For the irradiations an Aster BK (Assing SpA, Rome, Italy) medical radiography system is employed. Its X-ray tube has a rotating tungsten anode, its small focal spot was selected (0.6 mm) while its inherent filtration is equivalent to 3 mm Al.

Consistent with the IEC protocol, X-rays of quality RQA-5 were employed throughout the experiments. This means a high voltage of 70 kVp, a half-value layer of 6.8 mm and an additional Al filtration of 21 mm. For this purpose, a plate of Al type 1100 was used, with a purity of 99%. The Al plate is fixed as close as possible to the focal spot, i.e., adjacent to the collimator’s output window. The distance between the focal spot and the detector surface (source to detector distance-SDD) is kept at 1.5 m throughout the experiments { XE “source to detector distance:SDD” }.

#### 2.5.2. Assessment of STP

The response function or STP of the detector is the mean pixel value (MPV) as a function of the detector air kerma (DAK) { XE “detector air kerma:DAK” } on its entrance surface. In order to obtain the DAK, an RTI Piranha X-ray dose meter (RTI Group, Sweden) was positioned in place of the detector, while the same geometry retained as before. The IEC 62220-1:2003 standard recommends that a 4 mm thick lead foil is placed at 450 mm behind the dosimeter. In this way, the backscatter is reduced to <0.5% [21].

Then, a sequence of flat-field images at six exposure levels is acquired. Each exposure level consisted of five repetitions for averaging reasons.

For the MPV, a region of interest (ROI) { XE “region of interest:ROI” } of 100 × 100 pixels in the center of the exposed area was sampled and the values were averaged. Eventually, the system’s response curve was fitted using a linear equation of the following form [29,30]:

(1) $$MPV=a\cdot K_{a}+b,$$

where a is the detector’s gain factor (G) and b is the pixel offset at zero DAK (no radiation).

Under this setup, flat-field images were acquired in a range of exposures, each one in five repetitions for averaging.

#### 2.5.3. Assessment of MTF

Of the several methods that have been proposed for the practical determination of MTF, the slanted edge technique is followed in the present work. This procedure involves the use of a sharp edge tool of tungsten or lead. The PTW tungsten edge tool (PTW GmbH, Germany) was used for the experiments, which consists of a 1 mm thick tungsten edge plate (100 × 75 mm^2^) fixed on a 3 mm thick lead plate. The edge is placed on the carbon-fiber window of the detector. The tungsten edge device is slightly rotated so as to assume a shallow angle (1.5°–3°) with respect to the pixel matrix.

An image was acquired at a representative exposure level. From this image, the pixel values were derived and then converted back to input exposure levels via the inverse response function (linearization process).

The linearized pixel value as a function of the distance from the edge is the edge response function or edge spread function (ESF); this is repeated for a number of consecutive pixel lines (N, as defined by the IEC standard) and thus results in the oversampled ESF { XE “edge spread function:ESF” }. This ESF is differentiated with a kernel of [–1, 0, 1] or [–0.5, 0, 0.5] in order to derive the oversampled line spread function (LSF) { XE “line spread function:LSF” } across the edge. Subsequently, the MTF results are obtained by Fourier-transforming the oversampled LSF and considering its modulus. Its values are normalized at zero spatial frequency.

From the MTF curve, one can derive the limiting resolution of the system under investigation. It is defined as the spatial frequency where the MTF reaches 10% [31]. The spatial frequency where the MTF level reaches 50% is also used in the literature as a comparison measure between detectors [29]. The above will be noted, respectively, as MTF(10) and MTF(50) for brevity.

#### 2.5.4. Assessment of NNPS

In order to obtain the two-dimensional (2D) NPS, one has to acquire a flat-field image at a representative detector air kerma (DAK) level. On the acquired image, several ROIs are considered, each one with a 256 × 256 pixel area. Once the first ROI has been defined, the consecutive ones are drawn in series horizontally and vertically so that they overlap by 128 pixels in each direction.

Next, the IEC protocol suggests that detrending is performed. This is carried out by calculating a fitting 2D second-order polynomial function, S(x_i_,y_i_), to the linearized values of every image, then subtracting this function S(x_i_,y_i_) from the previous linearized values.

For every one of the defined ROIs, the 2D Fourier transform is calculated. The working equation according to the adopted IEC standard is as follows:

(2) $$W_{out}\left(u_{n},v_{k}\right)=\frac{\Delta x\Delta y}{M\cdot 256\cdot 256}\overset{M}{\underset{m=1}{\sum }}{\left[\overset{256}{\underset{i=1}{\sum }}\overset{256}{\underset{j=1}{\sum }}\left(I\left(x_{i},y_{j}\right)-S\left(x_{i},y_{j}\right)\right)\cdot exp\left(-2\pi i\left(u_{n}x_{i}+v_{k}y_{j}\right)\right)\right]}^{2},$$

where ΔxΔy is the pixel pitch, horizontally and vertically, respectively; M is the number of ROIs; I(x_i_,y_j_) denotes the linearized values; and S(x_i_,y_i_) is the fitting function (2D second-order polynomial).

The NPS normalized to the square of the large-area signal yields the normalized NPS (NNPS).

#### 2.5.5. Assessment of DQE

The DQE is calculated as a function of the spatial frequency by Equation (3).

(3) $$DQE\left(f\right)=\frac{SNR_{out}^{2}}{SNR_{in}^{2}}=\frac{MTF^{2}\left(f\right)}{q\cdot K_{\alpha }\cdot NNPS\left(f\right)},$$

where q is the fluence per exposure ratio, which equals the number of photons per unit area per exposure; K_a_ is the air kerma (kinetic energy released per unit mass) { XE “kinetic energy released per unit mass:kerma” }.

According to the IEC 2003 standard, for the RQA-5 beam quality, q equals 30,174 photons∙µGy^−1^∙mm^−2^ [21].

The IEC standard mandates that the DQE of a detector characterization should be reported starting from 0.5 cycles/mm. The value at zero spatial frequency is excluded because the DQE can be erroneously underestimated. This happens because at zero frequency, the NNPS can be unusually high because of low-frequency artifacts. NNPS is in the denominator of Equation (3), and a high value will lower DQE [29].

For the determination of the above metrics, the COQ plugin (Version 2.6) for the ImageJ suite (Version 1.52a) was used [32]. It performs the calculations according to the IEC standard 62220-1:2003.

## 3. Results

As soon as the fabricated screens were extracted from the silicone mold, they had a slightly flexible structure. The lack of brittleness facilitates removal from the mold without damage.

**Figure 3:**
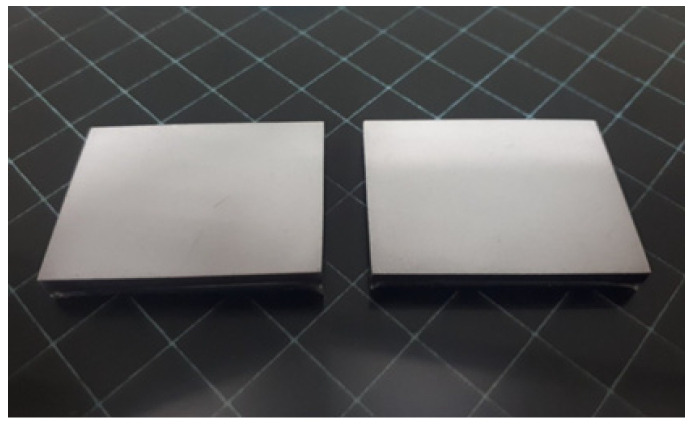
Finished screen samples. The dimensions are 36 × 27 mm^2^.

The bottom side of the screens, the active one that will be in contact with the CMOS, exhibits a uniformly coated surface (Figure 3). The upper side is dark gray due to the graphite content. The active surface has a smooth finish that resembles that of plastic or glass; the phosphor powder is enclosed within the resin and is not exposed to external factors. This is a distinct advantage of the method, since the resulting screens can be handled effortlessly.

### 3.1. Microscopic Properties

The fabricated resin screens were examined using electron microscopy. Their morphology characteristics were determined from the micrographs acquired. They were compared to corresponding micrographs of the commercial Min-R 2190.

A clear cross-section of the samples was cut with a lancet and the surface was ground manually with a suitable abrasive paper of P1200 grade. Nevertheless, access to a special disk-polishing instrument with dispenser was not possible; therefore, any debris visible in the sample micrographs are attributed to the imperfect manual polishing and the lack of the special grinding liquid.

**Figure 4:**
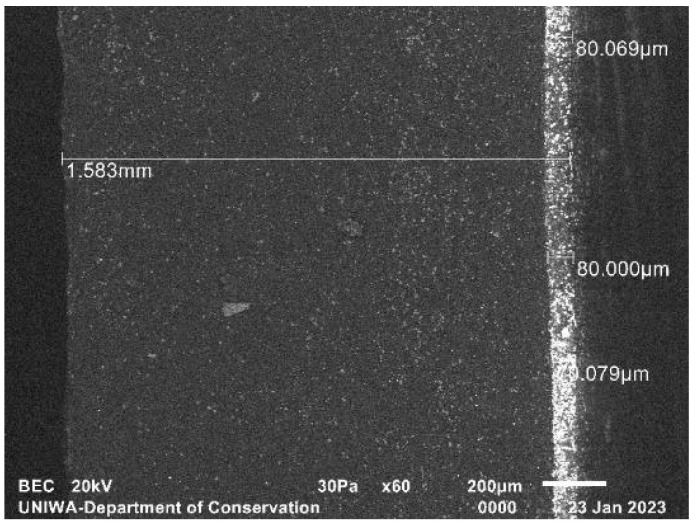
SEM micrograph of the fabricated 34 mg/cm^2^ screen.

Figure 4 illustrates the SEM micrograph of the 34 mg/cm^2^ screen prepared with the novel method. The phosphor grains are clearly packed towards the bottom resin layer. The phosphor layer has a measured thickness of ~80 µm and the total screen thickness is ~1.6 mm.

**Figure 5:**
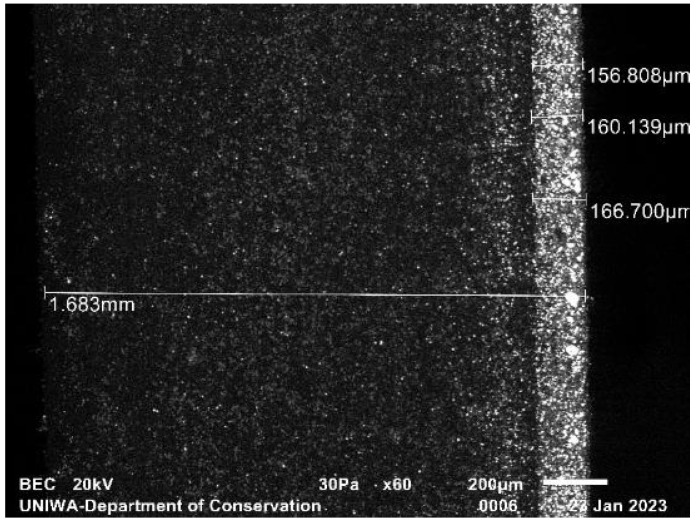
SEM micrograph of the fabricated 70 mg/cm^2^ screen.

Figure 5 reproduces the SEM micrograph of the higher surface density of the fabricated sample, 70 mg/cm^2^. Similarly to the previous sample, the phosphor grains remain accumulated towards the bottom of the resin tile. The phosphor layer has a measured thickness of ~160 µm and the total screen thickness is ~1.7 mm.

**Figure 6:**
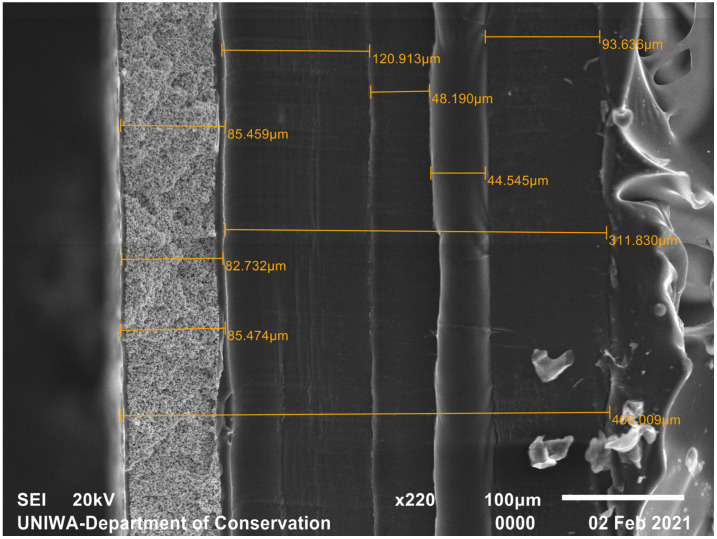
SEM micrograph of Min-R screen; 33.91 mg/cm^2^.

Indicatively, the SEM micrograph of the Min-R screen cross-section is also reproduced in Figure 6. Its surface density is reported to be 33.91 mg/cm^2^ [33,34,35]. The active layer has a thickness of ~83 µm, while several other thinner layers are visible, apparently light-absorbing and supportive ones. It is noted that, macroscopically, the phosphor side of the screen is white and the opposite side is black.

The dimensions derived from the images above are summarized in Table 1, along with another screen of wide commercial use, Lanex Medium, whose surface density approaches that of the 70 mg/cm^2^ fabricated sample. The two commercial screens’ values are reported by Cho et al. [33].

### 3.2. 70 mg/cm^2^ Screen Performance

The performance results of the fabricated resin screen are compared to a previously published CMOS detector [36]. This consists of the same CMOS sensor Remote RadEye HR coupled to a Gd_2_O_2_S:Eu scintillator screen that was fabricated with the classic sedimentation technique. The screen has a surface density of 65.1 mg/cm^2^, which is nearly the same as the fabricated screens of the present work. It is evaluated at the same conditions as the present work, i.e., medical radiography X-ray spectra of RQA-5 beam quality, as per the IEC standard 62220-1:2003.

#### 3.2.1. STP

**Figure 7:**
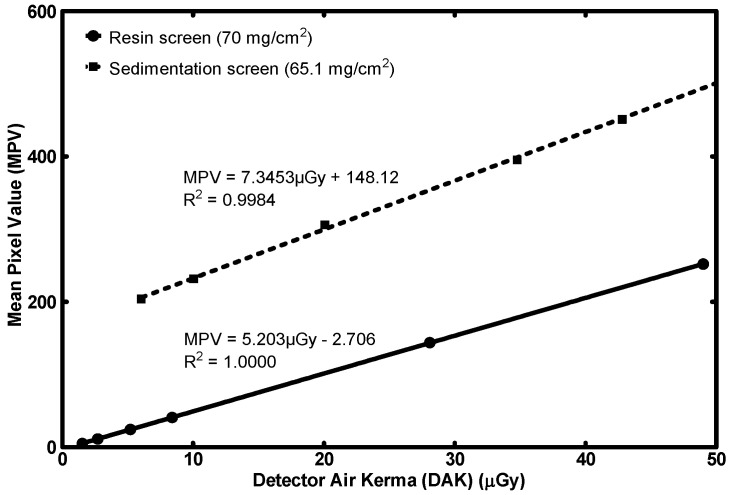
STP response comparison of the fabricated resin screen to a previously published sedimentation screen (high surface densities) [36].

Figure 7 includes the response of the 70 mg/cm^2^ resin screen along with the 65.1 mg/cm^2^ sedimentation screen. The MPV is plotted with the DAK value for six exposure levels and the linear interpolation is calculated for both detectors.

Both detectors’ coefficients of determination exceed 0.99 and therefore fulfill the IEC standard’s requirement for linearity. The two gain factors are approximately equal.

#### 3.2.2. MTF

**Figure 8:**
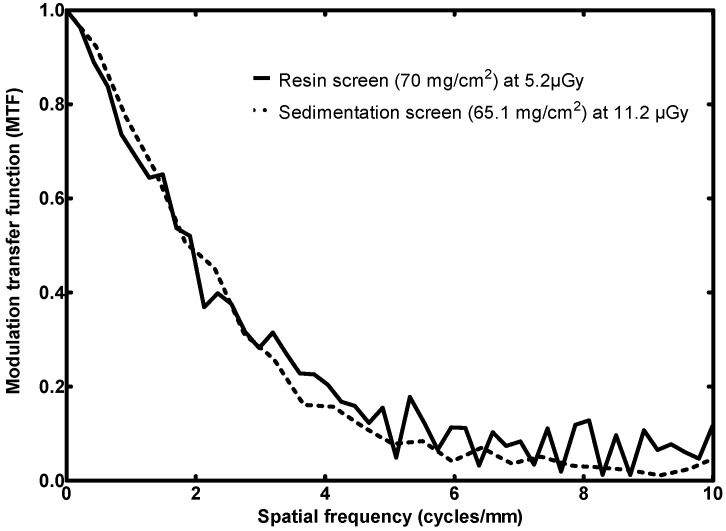
MTF response comparison of the fabricated resin screen to a previously published sedimentation screen (high surface densities) [36].

The MTF curves of the detectors are depicted in Figure 8. The same exposures were respected for the determination of MTF, NNPS, and DQE in order to ensure valid comparison, with 5.2 µGy (7.1 mAs for the present X-ray system).

The screen fabricated with the new resin method attains an MTF(50) of 1.9 cycles/mm and its limiting resolution, i.e., MTF(10), is 5.4 cycles/mm. As far as the Gd_2_O_2_S:Eu is concerned, its MTF(50) is 1.9 cycles/mm and MTF(10) is 4.7 cycles/mm.

#### 3.2.3. NNPS

**Figure 9:**
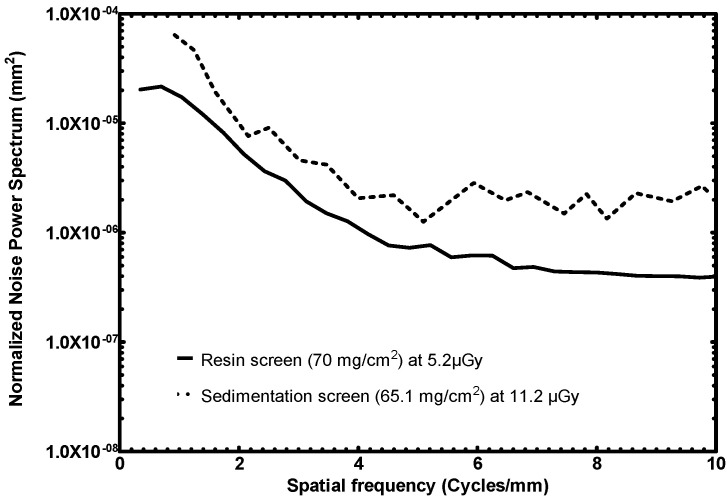
NNPS response comparison of the fabricated resin screen to a previously published sedimentation screen (high surface densities) [36].

In Figure 9, the NNPS response of the two screens is illustrated. It appears that the fabricated resin screen has 4–5 times lower noise than the sedimentation one.

It should be noted that the CMOS and Gd_2_O_2_S:Eu combination was irradiated at a greater exposure, more than double than that of this work’s fabricated detector. NNPS depends on radiation exposure. With increasing radiation exposure, the NNPS has the tendency to decrease. This happens because of the Poisson distribution in the detection of input X-ray quanta; with increasing exposure, the signal increase exceeds the noise increase [29]. Therefore, the fabricated detectors should exhibit higher noise spectra because of the lower exposure of the measurement; however, the opposite is confirmed, in favor of the novel method.

#### 3.2.4. DQE

**Figure 10:**
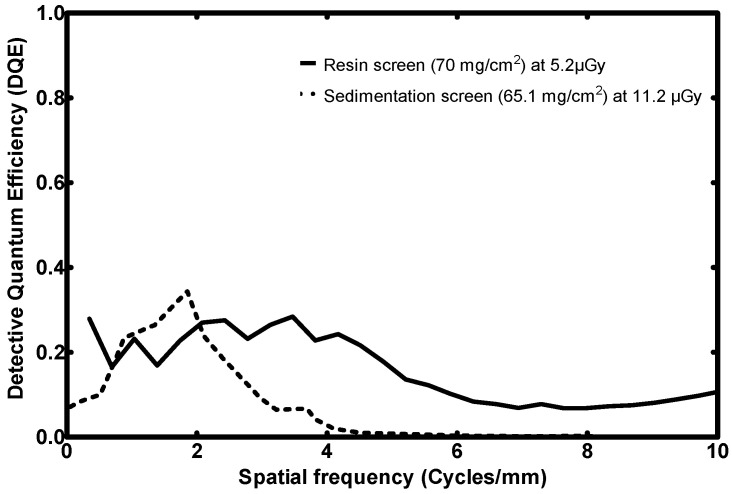
DQE performance comparison of the fabricated resin screen to a previously published sedimentation screen (high surface densities) [36].

Figure 10 is a comparison graph of DQE of the 70 mg/cm^2^ fabricated screen, along with that of the Gd_2_O_2_S:Eu screen.

The two detectors attain similar highest values (29% for the resin and 34% for the sedimentation method), yet at different spatial frequencies (1.9 cycles/mm for the Gd_2_O_2_S:Eu and 3.5 cycles/mm for the resin screen).

Most importantly, the proposed novel screen has a DQE 10–24% greater than that of its counterpart for spatial frequencies exceeding 2.7 cycles/mm. It even preserves a DQE ~10%, beyond 4.5 cycles/mm where the sedimentation screen’s DQE practically zeroes.

### 3.3. 34 mg/cm^2^ Screen Performance

The 34 mg/cm^2^ screen fabricated with the resin method is compared to the commercial Min-R 2190 [28], which has nearly the same surface density.

#### 3.3.1. STP

**Figure 11:**
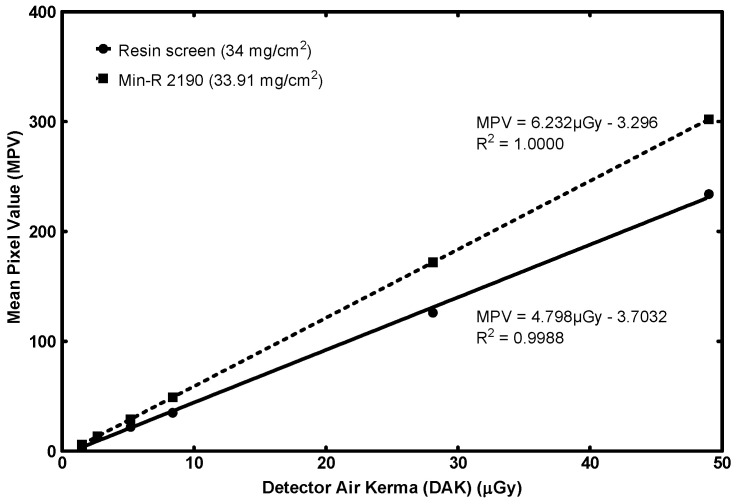
STP response comparison of the fabricated resin screen to a commercial screen (low surface densities).

Figure 11 contains the signal transfer property comparison for the two screens. The MPV is plotted with the DAK value for six exposure levels and the linear interpolation is calculated.

Both coefficients of determination are R^2^ ≥ 0.99; therefore, the IEC standard’s requirement is fulfilled.

The gain factor (G = 4.796 MPV/µGy) is lower than that of Min-R. As in the case of the 70 mg/cm^2^ detector above, this is due to the presence of graphite powder. Graphite absorbs a fraction of the stray light directed upwards, that would normally return and contribute to a higher signal (yet, at locations distant from the originating one, giving rise to image blur). On the other hand, the spatial response of the detector is positively influenced, as seen in the following sections.

#### 3.3.2. MTF

**Figure 12:**
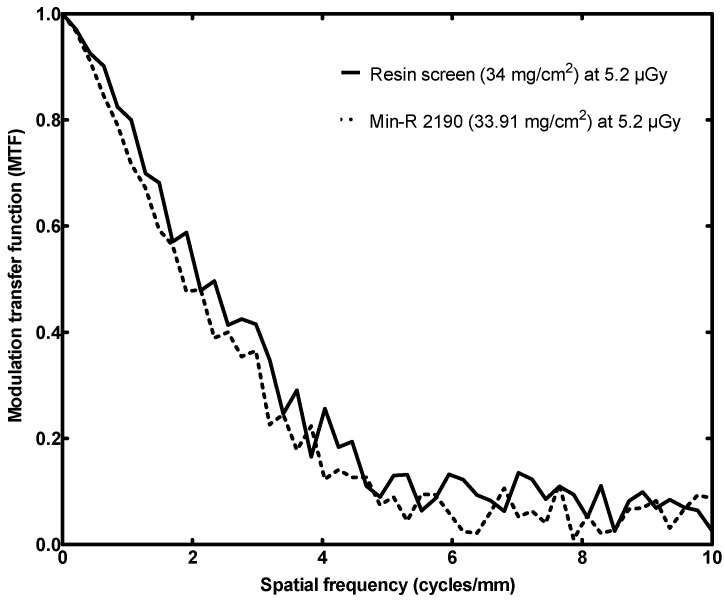
MTF response comparison of the fabricated resin screen to a commercial screen (low surface densities).

The MTF curve is illustrated in Figure 12. The image with the tungsten edge device for the determination of MTF was acquired at 5.2 µGy, i.e., 7.1 mAs, so that direct comparison to the CMOS and Min-R combination is possible. The same exposure was used for the determination of NNPS and the DQE, as above.

The MTF response falls to the 50% at the spatial frequency of 2.2 cycles/mm, as opposed to the 1.8 cycles/mm of the CMOS and Min-R combination. The limiting resolution of the detector is 5.3 cycles/mm and this is greater than the 4.5 cycles/mm of the commercial screen pair.

Practically, the proposed screen is able to discriminate details at a 0.8 cycles/mm greater spatial frequency. Actually, its MTF is higher in the whole examined range of spatial frequencies.

#### 3.3.3. NNPS

**Figure 13:**
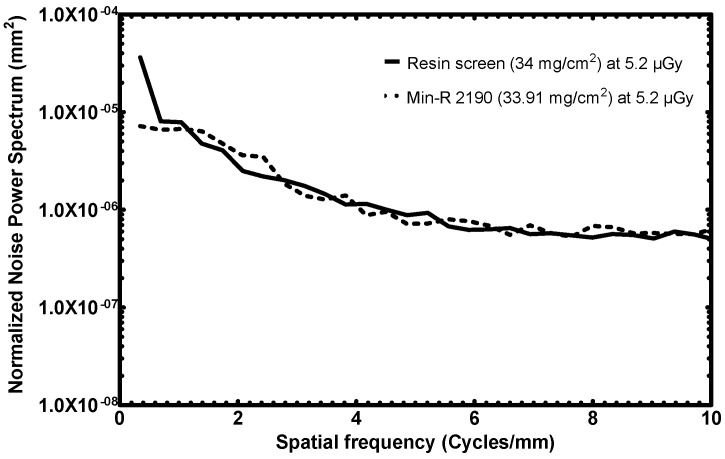
NNPS response comparison of the fabricated resin screen to a commercial screen (low surface densities).

The following Figure 13 is the NNPS response of the resin screen together with the CMOS and Min-R 2190 screen. The noise spectra of the two detectors rest at the same levels.

It is observed that the NNPS of the resin screen at the lowest spatial frequency, 0.3 cycles/mm, is quite high and reaches ~3.6∙10^−5^ mm. However, this could be an artifact because of remnant low-frequency trends—the same reason why the DQE(0) is excluded from the calculations, as is suggested by the IEC standard [21,29].

#### 3.3.4. DQE

**Figure 14:**
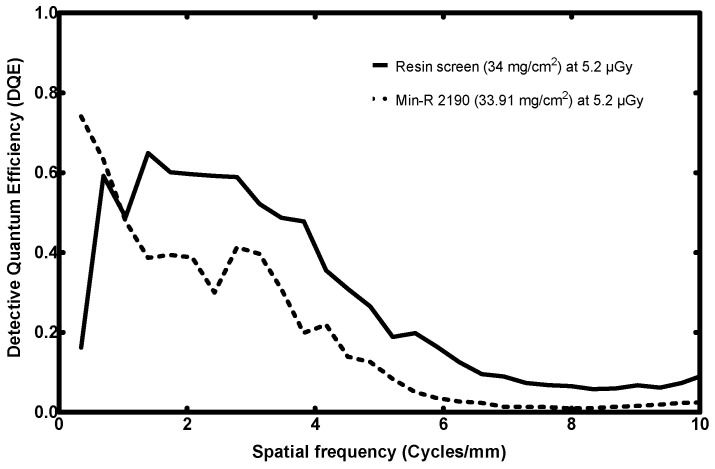
DQE response comparison of the fabricated resin screen to a commercial screen (low surface densities).

The DQE curve comparison is presented in Figure 14.

The fabricated screen’s DQE starts from relatively low values: 65% at 0.3 cycles/mm. This is expected since the NNPS exhibited quite a high value at this spatial frequency. As is explained above, this is a consequence of a low-frequency artifact.

Subsequently, the DQE promptly recovers, and at 0.7 cycles/mm, it attains similar levels to the Min-R combination, i.e., ~60%. From this spatial frequency onwards, the resin screen clearly outperforms the CMOS and Min-R combination. Its DQE remains 15–20% higher than that of the Min-R detector in the spatial frequency range from 1.4 to 4.5 cycles/mm. The proposed detector even exhibits 30% greater DQE than Min-R at 2.5 cycles/mm. After 4.5 cycles/mm, the resin screen remains steadily 10% higher than the other one.

The maximum DQE attained for the novel screen is 65% at 1.4 cycles/mm, while for the Min-R it is 74% at 0.4 cycles/mm.

The main results of the screens’ comparison are summarized in Table 2.

### 3.4. Sample X-ray Images

**Figure 15:**
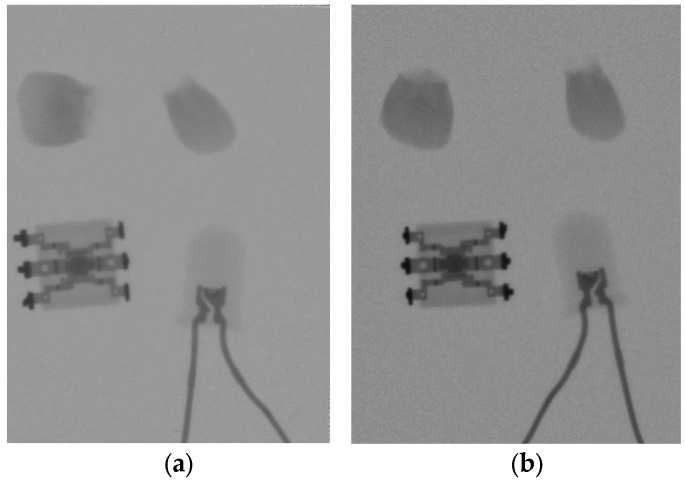
Sample X-ray images for comparison: (**a**) resin screen 34 mg/cm^2^; (**b**) Min-R 2190. Acquisition parameters: 70 kVp; 63 mA; 1 s; 20 mm Al filter; offset and gain correction. Setting: teeth, optocoupler, LED. The dimensions of the X-ray images are 1200 × 1600 pixels, corresponding to an active area of 27 × 36 mm^2^ [12].

In order to demonstrate the fabricated screens’ performance in a real scenario, below are reproduced sample images acquired at the same conditions for different screens coupled to the same CMOS sensor. In Figure 15a, the CMOS sensor is coupled to the 34 mg/cm^2^ screen of the novel method, though in Figure 15b, the commercial Min-R 2190 is used. The acquisition parameters were as follows: 70 kVp; 63 mA; 1 s; 20 mm Al filter; offset and gain correction.

Fine details of structures are visible in both X-ray images. According to Table 2, the resin screen attains a limiting resolution of 5.3 cycles/mm, which is at a similar level as that of the commercial screen. This is also true for the MTF and DQE levels, and this is confirmed by the X-ray image acquired using the fabricated resin screen (Figure 15a). Fine structures such as the internal copper connections of an integrated circuit (with dimensions 6.7 × 6.1 mm^2^), as well as those of an LED (housing diameter 5 mm) are clearly discernible, similar to the X-ray image of the commercial Min-R screen. The two detectors are able to reproduce the following:

- “Soft” elements (i.e., of low atomic number), such as the plastic housing of a 5 mm LED. Plastics in general are composed of hydrogen, oxygen, nitrogen, sulphur, chlorine and have atomic numbers between 5.4 and 8.4.
- “Medium” ones (i.e., of higher atomic number), such as the calcium of teeth. Calcium has an atomic number of 20.
- “Hard” elements (i.e., with the highest atomic number), such as the metallic connectors. Copper’s atomic number is 26.

## 4. Discussion

The resulting resin screen has an active surface with a smooth finish that resembles that of plastic or glass. The phosphor powder is enclosed within the resin and remains protected from external factors. This is a distinct advantage of the novel method, since the screen can be handled effortlessly.

Lack of brittleness and the protected phosphor greatly enhance the range of applications of the fabricated screens. They can be utilized in environments where the detector is exposed to mechanical shocks or humidity, for example. A detailed investigation of the life span of the newly fabricated screens has yet to be organized. However, blocks of resin prepared using the same ratio of components as the current experiments, and without any phosphor or graphite powder, have shown a substantial durability over the course of 4 years in our laboratory.

From Table 1, it is evident that there is a particularly good agreement between the fabricated screens of the present work with commercial ones of widespread use, at the respective surface densities. This is a proof that with the proposed method and preparation protocol, the same level of packing density of the scintillator powder can be achieved within the active layer of the produced screens.

The proposed screens’ coefficients of determination exceed 0.99 and therefore fulfill the IEC standard’s requirement for linearity. For the 70 mg/cm^2^ variant, the gain factor is approximately equal to the reference screen. The 34 mg/cm^2^ variant achieves a lower gain factor than its counterpart and this is attributed to the presence of graphite. However, the spatial response of the fabricated is positively influenced, as is obvious from the other image quality metrics.

In terms of MTF, the performances of the two 70 mg/cm^2^ screens are directly comparable. From zero up to 3.0 cycles/mm their MTF is similar. From 3.0 cycles/mm onwards, the proposed screen outperforms, having a roughly 5% greater MTF. Their limiting resolutions have a difference of 0.7 cycles/mm. ‘The MTF performances are comparable, despite the fact that the Gd_2_O_2_S:Eu screen has a slightly lower surface density and therefore it would be expected to promote resolution in place of efficiency. Most importantly, the limiting resolution of the 34 mg/cm^2^ screen is 5.3 cycles/mm and this is greater than the 4.5 cycles/mm of its counterpart, i.e., the commercial screen.

In the NNPS comparison of the 70 mg/cm^2^ screens, the novel resin screen exhibits 4–5 times lower noise than the sedimentation one. This level of performance was achieved despite the fact that the sedimentation screen was measured at more than double the exposure of the resin one, something that would be expected to result in lower noise spectra. The 34 mg/cm^2^ screens demonstrate similar noise levels.

In terms of the DQE, the proposed 70 mg/cm^2^ fabricated screen has a DQE 10–24% greater than that of its counterpart, for spatial frequencies exceeding 2.7 cycles/mm.

For the proposed 34 mg/cm^2^ resin screen, its DQE remains 15–20% higher than that of the Min-R between 1.4 and 4.5 cycles/mm. The proposed screen even exhibits a 30% greater DQE than Min-R at 2.5 cycles/mm, while after 4.5 cycles/mm its DQE remains steadily 10% higher.

## 5. Conclusions

The fabricated scintillation screens for X-ray imaging reported in this paper exhibited comparable or even better performance in widely accepted image quality metrics that were determined according to international standard procedures. Specifically, the thin screen achieved a DQE 15–20% greater, on average, than its counterpart, while its limiting resolution was 0.8 cycles/mm better. Similarly, the thick screen achieved a DQE 10–24% greater than its own counterpart, and its limiting resolution was improved by 0.7 cycles/mm.

The screens fabricated with our method exhibit resistance to strains caused by handling. The scintillator powder remains enclosed in a resin envelope that protects it while offering enhanced rigidity of the screen as a piece. In contrast, the borosilicate glass or fused silica of the classic sedimentation method is several times thinner and more fragile in comparison to a resin tile of 1–2 mm thickness.

The valuable scintillating material is used almost in its entirety. Only an imperceptible amount is lost. In contrast, in the sedimentation technique, the material loss can amount up to 78%.

The technique does not demand sophisticated equipment and particular expertise to run. This can lower costs dramatically in comparison to other techniques. Simplicity can enable individual researchers and small facilities to obtain thin powder screens for scientific or industrial applications.

## Figures and Tables

**Table 1:** Screens’ physical parameters.

Phosphor Screen	Surface Density (mg/cm^2^)	Phosphor Layer Thickness (µm)
Resin screen (SEM Figure 4)	34	80
Resin screen (SEM Figure 5)	70	160
Min-R 2190 (Figure 6)	33.91	85
Lanex Medium	59.20	160

**Table 2:** Characterization summary.

Screen	Gain Factor (MPV/µGy)	Coefficient of Determination R^2^	MTF(50) @ 5.2 µGy (cycles/mm)	MTF(10) @ 5.2 µGy (cycles/mm)	DQE max @ 5.2 µGy (% @ cycles/mm)
Resin 70 mg/cm^2^ Resin 34 mg/cm^2^ Gd_2_O_2_S:Eu	5.203	1.000	1.9	5.4	29% @ 3.5
	4.798	0.999	2.2	5.3	65% @ 1.4
	7.345	0.998	1.9 *	4.7 *	34% @ 2 *
Min-R	6.232	1.000	1.8	4.5	74% @ 0.4

* Measured at 11.2 µGy.

## Data Availability

Data are contained within the article.
